# Resveratrol as a potential natural compound to ameliorate cognitive impairment in aluminum-exposed mice: impacts on behavior, purinergic system, and brain inflammation

**DOI:** 10.1007/s11011-026-01859-z

**Published:** 2026-05-19

**Authors:** Alice Estivalet Visentini, Karine Paula Reichert, Maria Rosa Chitolina Schetinger, Nathieli Bianchin Bottari, Vanessa Valéria Miron, Milagros Fanny Vera Castro, Marcylene Vieira da Silveira, Charles Elias Assmann, Adriel Antonio Schirmann, Vera Maria Melchiors Morsch

**Affiliations:** PostGraduate Program in Toxicological Biochemistry, Department of Biochemistry and Molecular Biology, Federal University of Santa Maria (UFSM), Santa Maria, Rio Grande do Sul Brazil

**Keywords:** Neurodegeneration, Antioxidant, Alzheimer’s disease, Behavior assessment, BDNF

## Abstract

Aluminum (Al³⁺) accumulates in the brain and has been linked to neurodegeneration. When combined with citrate, its absorption increase, raising the risk of neurodegenerative disorders such as Alzheimer’s disease. Resveratrol (RSV), a polyphenol with anti-inflammatory, antioxidant, and neuroprotective properties, has been associated with aging prevention by reducing oxidative stress. This in vivo study aimed to determine whether RSV could protect male Swiss mice from Al³⁺-induced cognitive impairment, alterations in purinergic signaling, and brain inflammation. The study was conducted using 60 animals treated with aluminum chloride (AlCl₃ 50 mg/Kg, gavage), citrate (AlCl₃ 50 mg/Kg + CIT 100 mg/Kg, gavage), and resveratrol (AlCl₃ 50 mg/Kg + RSV 100 mg/Kg, gavage), along with their respective Controls, for 30 days every 48 h. Behavioral assessments included the open field test, object recognition test, and Y-maze test to evaluate cognitive performance. Enzymatic activity of purinergic pathways and levels of receptors and inflammation-related molecules were also analyzed. AlCl₃ and AlCl₃ + CIT induced an inflammatory response in mice, as evidenced by the modulation of purinergic system enzymes associated with inflammation. Al³⁺ intoxication also triggered behavior alterations, which was modulated by CIT and alleviated by RSV treatment. Western blot findings revealed that AlCl₃ and AlCl₃ + CIT altered protein levels of purinergic receptors (A1R, A2AR, and P2 × 7R), the inflammasome protein NLRP3, the pro-inflammatory cytokine IL-1β, and the neurotrophic factor BDNF. RSV counteracted the toxic effects of Al³⁺, providing neuroprotection against its toxicity and presenting itself as a potential therapeutic candidate for cognitive deficits.

**Supplementary Information:**

The online version contains supplementary material available at 10.1007/s11011-026-01859-z.

## Highlights

RSV counteracts Al^3+^-induced effects in purinergic enzymes and receptors of the cerebral cortex.

The addition of citrate altered the toxicity of Al³⁺.

RSV has neuroprotective effects against Al^3+^-induced toxicity.

**Graphical abstract:**

**Supplementary Information:**

The online version contains supplementary material available at 10.1007/s11011-026-01859-z.

## Graphical abstract

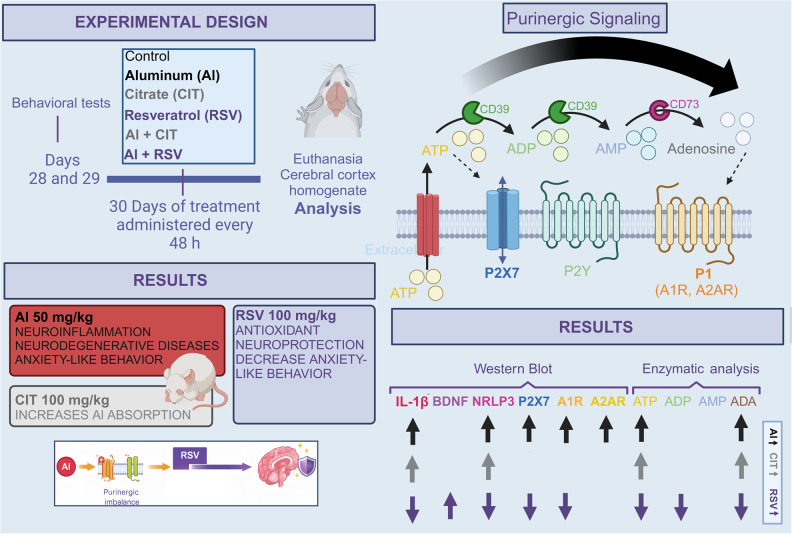

## Introduction

Aluminum (Al^3+^) is a trivalent metal with recognized neurotoxic potential widely used in industry due to its physicochemical properties. One of its main uses is in the treatment of drinking water, in the form of sulfate (Al₂(SO₄)₃); thus, humans are constantly exposed to this element. Al^3+^ can bioaccumulate in tissues and organs, especially in the brain, as it crosses the blood–brain barrier (BBB) (Lukiw et al. 2018). Thus, research has suggested that Al^3+^ triggers neuroinflammatory effects and may be associated to the development of neurodegenerative diseases, such as Alzheimer’s disease (AD) (Exley 2013; Bondy 2016). At the same time, broader toxicological evaluations indicate that aluminum exposure is linked to a wide spectrum of biological effects and health outcomes, and that extrapolating findings from experimental models directly to complex human neurodegenerative diseases requires caution (Crisponi et al. 2013). Accordingly, the relationship between aluminum exposure and AD remains controversial. Recent comprehensive reviews emphasize that, although aluminum clearly induces neurotoxic, pro-oxidative, and pro-inflammatory effects in experimental models, current epidemiological and clinical evidence does not support a direct causal role of aluminum as a primary etiological factor in AD. Instead, aluminum is more appropriately regarded as a potential contributory or modulatory factor that may influence neurodegenerative processes under specific conditions (Colomina and Peris-Sampedro 2017; Kawahara et al. 2021). Notably, aluminum toxicokinetics are strongly influenced by chemical speciation. In the presence of citrate (CIT), aluminum forms stable and soluble Al–citrate complexes that prevent its precipitation as insoluble hydroxides in the gastrointestinal tract, thereby facilitating intestinal absorption, increasing systemic bioavailability, and enhancing tissue distribution (Greger et al. 1997; Nolan et al. 1990; Wu et al. 2012). This increased bioavailability may amplify aluminum accumulation and its neurotoxic effects, thereby enhancing neuronal and glial vulnerability and promoting neurotoxic outcomes that overlap with mechanisms relevant to AD, within a broader framework of aluminum-related toxicity rather than a disease-specific etiology (Crisponi et al. 2013; Colomina and Peris-Sampedro 2017).

AD is a debilitating chronic neurodegenerative disorder resulting from a multifactorial process that involves genetic and environmental components, with exposure to Al^3+^ being one of the most studied environmental risk factors (Kandimalla et al. 2016). Consistent with this multifactorial view, environmental agents such as aluminum are increasingly considered modifiers of disease susceptibility or progression, rather than isolated causative factors (Crisponi et al. 2013; Colomina and Peris-Sampedro 2017). According to the World Health Organization (WHO), more than 50 million people worldwide are affected by dementia, and will almost triple by 2050 (WHO, 2021). Among the main pathological characteristics of AD are the accumulation of β-amyloid (Aβ), tau hyperphosphorylation (p-tau), and aggregation of neurofibrillary tangles (NFTs), which lead to the loss of synaptic and neuronal function (Bloom, 2014). However, other mechanisms are also associated with the pathogenesis of AD.

In recent years, the purinergic system has emerged as a promising pathway in the context of AD and other neurodegenerative conditions (Burnstock 2017; Illes et al. 2019). The purinergic system is comprised of receptors, enzymes, and signaling molecules that participate in many biological functions (Huang and Reichardt, 2021). The receptors of the purinergic system can be divided into two main families: P2 and P1. The P2 family is further subdivided into the P2X and P2Y subfamilies. P2Y receptors are G protein-coupled receptors (GPCRs), while P2X receptors are ion channels activated by nucleotides. Among the subtypes of the P2X family, the P2 × 7R receptor has emerged as one of the most studied due to its role in inflammatory processes, apoptosis, and neurodegeneration. The P1 family, on the other hand, consists of four GPCR subtypes: A1R, A2AR, A2BR, and A3R, which are activated by adenosine (ADO). These receptors play essential roles in regulating neurotransmission, inflammation, and immune responses. For instance, the A1R receptor is associated with inhibiting neurotransmitter release, while A2AR is known to modulate inflammatory and synaptic processes, both of which are relevant in the context of neurodegenerative diseases such as AD. There are also enzymes responsible for the hydrolysis of nucleotides, including adenosine mono-, di-, and triphosphates (AMP, ADP, and ATP, respectively), converting them into nucleosides such as ADO. These enzymes are represented by ecto-nucleoside triphosphate diphosphohydrolase (NTPDase1; CD39), ecto-5’-nucleotidase (5′-NT; CD73), and adenosine deaminase (ADA), all expressed on the cell surface (Reichert et al. 2020; Huang and Reichardt, 2021). Recent studies suggest that aluminum (Al³⁺), a neurotoxin widely associated with neuroinflammation and AD, may interfere with the purinergic system. Evidence indicates that Al³⁺ can affect the expression and activity of enzymes such as CD39 and CD73, as well as the functionality of purinergic receptors like P2 × 7R, A1R, and A2AR. These effects may amplify neuroinflammation, oxidative stress, and neuronal apoptosis, contributing to the progression of neurodegeneration (Reichert et al. 2020). The modulation of the purinergic system, either through pharmacological interventions targeting receptors or by adjusting enzymatic activity, has been identified as a promising approach for AD treatment. Furthermore, understanding how Al^3+^ interacts with this system could open new perspectives for the prevention and management of neurodegenerative diseases. Recent evidence has also shown that Al^3+^ can alter parameters of microglial and embryonic neural progenitor cells, possibly by altering purinergic signaling components (Assmann et al. 2021; Reichert et al. 2020).

Extensive research has been conducted to identify natural compounds that can prevent and/or delay neurodegenerative diseases. Among these molecules, resveratrol, a polyphenol found in at least 72 plant species, with the highest concentrations present in the dried roots of *Polygonum cuspidatum* (Meng et al. 2020). Resveratrol has been widely reported to exert neuroprotective effects through its antioxidant and anti-inflammatory properties, as well as through the modulation of molecular pathways involved in neuroinflammation and synaptic function. Experimental evidence indicates that resveratrol negatively regulates the activation of the NLRP3 inflammasome and reduces the production of pro-inflammatory cytokines, including interleukin-1β (IL-1β), which are critically involved in neuroinflammatory responses and cognitive impairment (Chang et al. 2015; Rahman et al. 2020; Tufekci et al. 2021). In parallel, resveratrol has been shown to positively modulate brain-derived neurotrophic factor (BDNF), a key mediator of synaptic plasticity, neuronal survival, and memory processes. Since neuroinflammatory signaling pathways, including NLRP3 activation and IL-1β release, are known to suppress BDNF expression and impair cognitive function, the ability of resveratrol to attenuate inflammation while preserving neurotrophic support may represent a central mechanism underlying its neuroprotective effects in models of neurotoxicity and neurodegeneration. Furthermore, its neuroprotective, anti-amyloidogenic, and antifibrillary effects have been reported (Gomes et al. 2018). This suggests that RSV may act as an antioxidant, preventing the formation of toxic Aβ oligomers and AD-related protofibrillary intermediates (Ciccone et al. 2022). RSV also appears to protect against the dysregulation of brain energy homeostasis observed in AD models (Yang et al. 2021; Fracasso et al. 2021), although its neuroprotective mechanism is not yet fully understood.

Based on evidence that aluminum exposure promotes neuroinflammation and disrupts purinergic signaling, and that citrate can enhance aluminum bioavailability, the present study was designed to investigate whether chronic Al³⁺ exposure induces behavioral alterations and molecular changes related to purinergic and inflammatory pathways in the cerebral cortex. Given the reported antioxidant and neuroprotective properties of resveratrol, we hypothesized that RSV treatment would counteract aluminum-induced neurotoxicity by modulating purinergic signaling pathways that are functionally linked to neuroinflammation, synaptic regulation, and cognitive performance, attenuating neuroinflammatory responses, and preserving neurotrophic support. Accordingly, the aim of this study was to evaluate the effects of resveratrol on behavioral performance, purinergic enzyme activity and receptor expression, inflammatory markers, and BDNF levels in the cerebral cortex of male Swiss mice chronically exposed to Al³⁺, with or without citrate.

## Materials and methods

### Chemicals and reagents

RSV (C_14_H_12_O_3_; molecular weight = 228.25 g/mol; > 98% purity), aluminum chloride (AlCl_3_; molecular weight = 133.34 g/mol; >99% purity), dinitrophenylhydrazine (DNPH), nucleotides, D-glucose anhydrous, thiobarbituric acid (TBA), trichloroacetic acid (TCA), malondialdehyde (MDA), and malachite green were obtained from Sigma-Aldrich (St. Louis, MO, USA). All antibodies were obtained from Santa Cruz Biotechnology (Dallas, Texas, USA). All other chemical reagents were of analytical grade. All solutions were prepared with high purity Milli-Q water.

To avoid Al^3+^ contamination, only plastic materials were used during the experiments. All laboratory materials (beakers, pipette tips, volumetric flasks, etc.) were immersed for at least 48 h in a 10% HNO_3_/ethanol solution and washed with Milli-Q purified water before use.

### Animals

For this study, 60 male Swiss mice (45–60 days old, weighing 30–35 g), obtained from the Animal Reproduction Center of the Federal University of Santa Maria (Santa Maria, RS, Brazil), were used. The exclusive use of male mice was chosen to reduce biological variability associated with hormonal fluctuations of the estrous cycle in females, which can significantly influence behavioral and neuroinflammatory outcomes (Becker et al. 2005). This choice was based on criteria of experimental homogeneity and data reproducibility, particularly in models involving purinergic system analyses and behavior analyses, where hormonal variations may act as confounding factors (Gillies and McArthur 2010). Furthermore, this decision is in line with previous studies that employed male-only cohorts to evaluate aluminum-induced neurotoxicity (Al-Amin et al. 2016; Bottari et al. 2016). Before beginning the experiment, the mice underwent a 1-week adaptation period and were distributed at five mice per box (30 × 20 × 13 cm). The mice were maintained under standard conditions in an environment with a Controlled temperature of 25 ± 2 °C and relative humidity of 45% − 55%, 12 h light/dark cycle (lights on at 07:00 h). Both solid (commercial food) and water diets were provided *ad libitum*. All behavioral tests were conducted during the light phase, between 08:00 and 12:00 h, and always 24 h after the previous gavage administration, in order to minimize circadian influences and acute treatment-related effects. All procedures described were approved by the Ethics Committee on the Use of Animals of the Federal University of Santa Maria, Brazil, under registration number 5,043,091,121.

### Experimental design and treatments

**Fig. 1:**
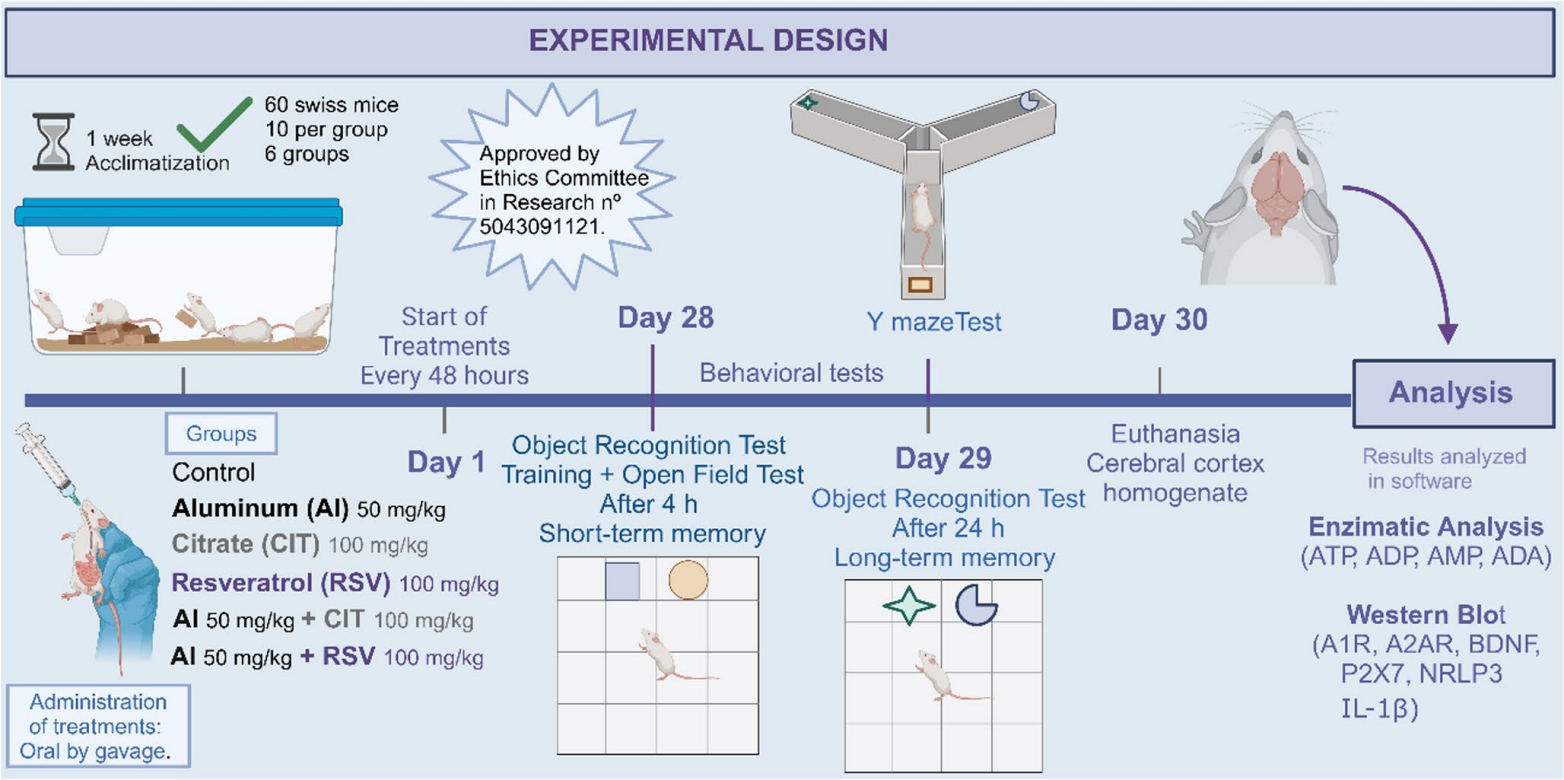
Experimental study design

After a 7-day adaptation period to housing conditions, the administration of all treatments was initiated. The mice received a solution containing AlCl₃ (50 mg/kg body weight) and/or RSV (100 mg/kg body weight), diluted in Milli-Q water and 0.1% dimethyl sulfoxide (DMSO), administered via oral gavage every 48 h for 30 days, based on previous studies demonstrating that these doses reliably induce aluminum-related behavioral and molecular alterations and allow the evaluation of resveratrol’s neuroprotective effects (Al-Amin et al. 2016; Bottari et al. 2016). In line with these established exposure models, the present study focused on functional, behavioral, and molecular endpoints to characterize central effects of aluminum and resveratrol, rather than on direct quantification of compound levels in biological matrices (Reichert et al. 2020; Assmann et al. (2021). The establishment of the aluminum-induced neurotoxicity model was assessed using a predefined set of behavioral, biochemical, and molecular indicators. Behavioral indicators included alterations in locomotor and exploratory activity and memory-related performance evaluated by the open field test, object recognition test, and Y-maze test. Molecular and biochemical indicators comprised changes in purinergic enzyme activities and purinergic receptor expression in the cerebral cortex, as well as modulation of inflammatory markers (NLRP3 and IL-1β) and the neurotrophic factor BDNF. Together, these parameters were used to confirm the induction of aluminum-related neurobehavioral and neuroinflammatory alterations. Resveratrol was dissolved in 0.1% DMSO in Milli-Q water; this concentration is widely used and considered behaviorally and physiologically inert in mice. DMSO was used exclusively as a solvent for resveratrol, and no experimental group received DMSO alone as a vehicle control. To enhance AlCl₃ absorption, citrate (CIT, 100 mg/kg body weight) was added to the solution and also administered via oral gavage every 48 h for 30 days, given its established role in increasing aluminum bioavailability through the formation of soluble Al–citrate complexes (Greger et al. 1997). All gavage administrations were performed at a volume of 10 mL/kg body weight. Behavioral assessments were conducted 24 h after the last gavage, ensuring that the observed effects reflected subchronic exposure rather than acute pharmacological or handling-related responses. The animals were randomly assigned to the following groups: Control (*n* = 10), saline solution; CIT (*n* = 10); AlCl₃ (50 mg/kg, *n* = 10); RSV (100 mg/kg, *n* = 10); AlCl₃ (50 mg/kg) + CIT (100 mg/kg, *n* = 10); and AlCl₃ (50 mg/kg) + RSV (100 mg/kg, *n* = 10). Randomization was performed using a simple random allocation procedure based on animal identification numbers prior to treatment initiation. Experimenters conducting behavioral tests and biochemical analyses were blinded to group allocation during data acquisition and analysis. On the final day of the experimental protocol, the animals were anesthetized in an isoflurane atmosphere and euthanized by decapitation. Their brains were carefully removed, and the cerebral cortex was isolated for further analysis (Fig. 1). While our model included an AlCl₃+CIT exposure arm, we did not include an AlCl₃+CIT + RSV combination arm; we acknowledge this as a limitation and prioritize it for future studies.

This figure shows the experimental protocol for the treatments with aluminum chloride (AlCl₃ 50 mg/kg, gavage), citrate (CIT 100 mg/kg, gavage), and resveratrol (RSV 100 mg/kg, gavage) in a model of aluminum intoxication in male Swiss mice. Over a period of 30 days, the compounds were orally administered to the mice every 48 h, who also underwent behavioral tasks, such as the open field test (OFT), object recognition test (ORT), and Y-maze test to assess cognitive performance. After euthanasia, the cerebral cortex was removed and used for biochemical analyses.

### Behavioral tests

#### Open field test (OFT)

The OFT was performed as described by Furlan and Brandão (1999). This test was used to assess locomotor activity and exploratory behavior. The test was conducted in a box measuring 45 × 45 cm. A mouse was placed in the center of the box and allowed to explore for 5 min. The parameters that were analyzed included the total number of entries and a representative trajectory plot. These parameters were recorded and later analyzed using the ANY-maze™ video tracking software (Stoelting, CO, USA, version 8.0).

#### Object recognition test (ORT)

The ORT was performed according to the protocol outlined by Sauvage et al. (2008), in a box measuring 45 × 45 cm. This test was used to evaluate recognition memory (discrimination index), including short-term and long-term memory performance. The test consisted of three sessions: a training session, followed by assessments of short-term memory (4 h after training) and long-term memory (24 h after training). During the training session, mice were introduced to two objects with the same size and shape but different colors, defined as familiar objects (F1 and F2), for 5 min (Lego toys). In the subsequent test session, a familiar object (F2) was replaced by a new object (N) 4 h after training; a Lego toy different from those used in the previous session was used. Then, the mice were allowed to explore the familiar object and new object for 5 min. At the beginning of each test, mice were placed in the center of the box and the time taken to explore each object was recorded. To facilitate localization, a crossover design was implemented on the floor of the box during all test sessions. The new and familiar objects were placed alternately to exclude potential preference for a particular spatial location of objects in the box. Exploration was defined as smelling or touching the object with the front paws when the mouse was within 2 cm of the objects (Ennaceur and Delacour 1988). The recognition time for each object was calculated for each mouse using the formula $\:{\left.\left(\:\frac{TF1}{TF1+TN}\right.\right)}^{}$× 100, where TF1 is the exploration time of the familiar object and TN is the exploration time of the new object, as described by Dias et al. (2007). Furthermore, the distance traveled, average speed, and immobility time were recorded and later analyzed using the ANY-maze™ video tracking software (Stoelting, CO, USA, version 8.0). The boxes used in the tests were cleaned with 70% alcohol between sessions.

#### Y-maze test

The Y-maze test was performed as described by Kraeuter et al. (2019). This test was used to assess spatial working memory and exploratory behavior. A mouse was placed at the center of a Y-shaped apparatus, with dimensions of 30 cm (length of each arm) × 5 cm (width) × 12 cm (wall height). The parameters measured included total distance covered (m) and number of arm alternations. Data were recorded and analyzed using the ANY-maze™ video tracking software.

### Purinergic enzyme activities

#### NTPDase and 5’-NT activities

NTPDase and 5’-NT activities were analyzed in the cortex following the methods described by Schetinger et al. (2000) and Heymann et al. (1984), respectively. Twenty microliters of sample were added to the reaction mixture for each enzyme and pre-incubated at 37 °C for 10 min with ATP or ADP (1mM) for NTPDase or AMP (2mM) for 5’-NT as substrate. The reactions were stopped by adding 10% trichloroacetic acid (TCA). The released inorganic phosphate was determined using malachite green as a colorimetric reagent and read at 630 nm. Specific enzyme activities are presented as nmol of Pi released per minute per mg of protein.

#### ADA activity

ADA activity was assessed based on the amount of ammonia produced, following the method of Giusti and Galanti (1984). Briefly, 50 µL of brain homogenate was reacted with 21 mmol/L ADO (pH 6.5) at 37 °C for 60 min. After the incubation period, the reaction was stopped by adding a solution containing 167.8 nM sodium nitroprusside, 106.2 mM phenol, and sodium hypochlorite. The amount of ammonia produced was quantified at 620 nm. The results are expressed as units of ADO per mg of protein.

### Protein concentration

Cortical tissue samples were homogenized in ice-cold phosphate-buffered saline (PBS) using a glass–Teflon homogenizer. The homogenates were then centrifuged at 10,000 × g for 10 min at 4 °C to remove cellular debris. The resulting supernatants were collected and used for subsequent biochemical and molecular analyses. Protein concentration was determined according to the method described by Bradford (1976), using bovine serum albumin as a standard.

### Western blot

The density of receptors was assessed using Western Blot as described by Rebola et al. (2003). In brief, the cortex was homogenized in ice-cold radioimmunoprecipitation assay (RIPA) buffer with 1 mM phosphatase and protease inhibitors and centrifuged at 12,000 rpm at 4 °C for 10 min. Subsequently, the proteins were separated using sodium dodecyl sulfate–polyacrylamide gel electrophoresis (SDS-PAGE) and transferred to Immun-Blot^®^ polyvinylidene difluoride (PVDF) membranes (Bio-Rad Laboratories, CA, USA). After blocking, membranes were incubated overnight at 4 °C with the following primary antibodies: P2X purinoceptor 7 (Rabbit; Santa Cruz Biotechnology; SC25698; diluted 1:500), Adenosine A2A receptor (Rabbit; Thermo; PA1042; diluted 1:800), Adenosine A1 receptor (Rabbit; Thermo; PA1041A; diluted 1:500), Brain-derived neurotrophic factor (Mouse; Santa Cruz; SC65513; diluted 1:500), NLR family pyrin domain containing 3 (Anti-NLR family pyrin domain containing 3 (NLRP3) (Rabbit; Thermo; PA5-79740; diluted 1:500) and Interleukin-1 beta (Anti-Interleukin-1 beta (IL-1β) (Rabbit; CusaBio; 005122; diluted 1:500). Following this step, the membranes were washed with Tris-buffered saline (pH 7.6) with 0.1% Tween 20 (TBST) and further incubated with anti-rabbit or anti-mouse secondary antibody (diluted 1:10,000) at room temperature for 90 min. The membranes were washed again, incubated with an enhanced chemiluminescent substrate (Immobilon^®^ Forte Western HRP Substrate, Merck KGaA, Darmstadt, Germany), and analyzed with an Amersham Imager 600 (GE, Healthcare Life Sciences, IL, USA) equipment. As a Control, membranes were reprobed with a β-actin antibody (diluted 1:1000). Densitometric analysis was performed using ImageJ software (NIH, USA). Protein band intensities were normalized to β-actin, which served as the internal loading control. Results are expressed as relative optical density values normalized to the Control group.

Due to the limited availability of cerebral cortex tissue following extensive behavioral, enzymatic, and biochemical analyses, Western blot experiments were conducted with a reduced number of biological replicates (*n* = 2–7 per group), consistent with previous in vivo neurotoxicity studies using mouse brain tissue (Ising et al. 2019; Bottari et al. 2019; Fracasso et al. 2021; Assmann et al. 2021; Zhu et al. 2023; Gan et al. 2025; Xu et al. 2026). These data were used to complement and support the functional and biochemical findings, rather than as standalone quantitative endpoints.

### Statistical analysis

Statistical analyses were performed using GraphPad Prism 8.0 (GraphPad Software, San Diego, CA, USA). Data distribution was initially assessed for normality using the Shapiro–Wilk test, and homogeneity of variances was evaluated using Levene’s test. No outliers were excluded unless justified by technical error during data acquisition. For datasets involving aluminum exposure and resveratrol treatment, data were analyzed using two-way analysis of variance (two-way ANOVA) with the following independent factors: (i) Aluminum exposure, with three levels (no aluminum, AlCl₃, and AlCl₃ + citrate), and (ii) Resveratrol treatment, with two levels (absence or presence). This approach allowed the evaluation of the main effects of each factor as well as their interaction (Aluminum × Resveratrol). When a significant main effect or interaction was detected, post hoc comparisons were performed using Tukey’s multiple comparisons test. Effect sizes were calculated using partial eta squared (η²p) for ANOVA analyses. Data are presented as mean ± standard error of the mean (SEM). Exact F values, degrees of freedom, and p values are reported in the Results section or figure legends. A significance level of *p* < 0.05 was adopted for all analyses. The complete raw numerical data used for all statistical analyses are provided in Supplementary Table S1.

## Results

### RSV ameliorates the AlCl_3_-induced behavioral changes in male mice

**Fig. 2:**
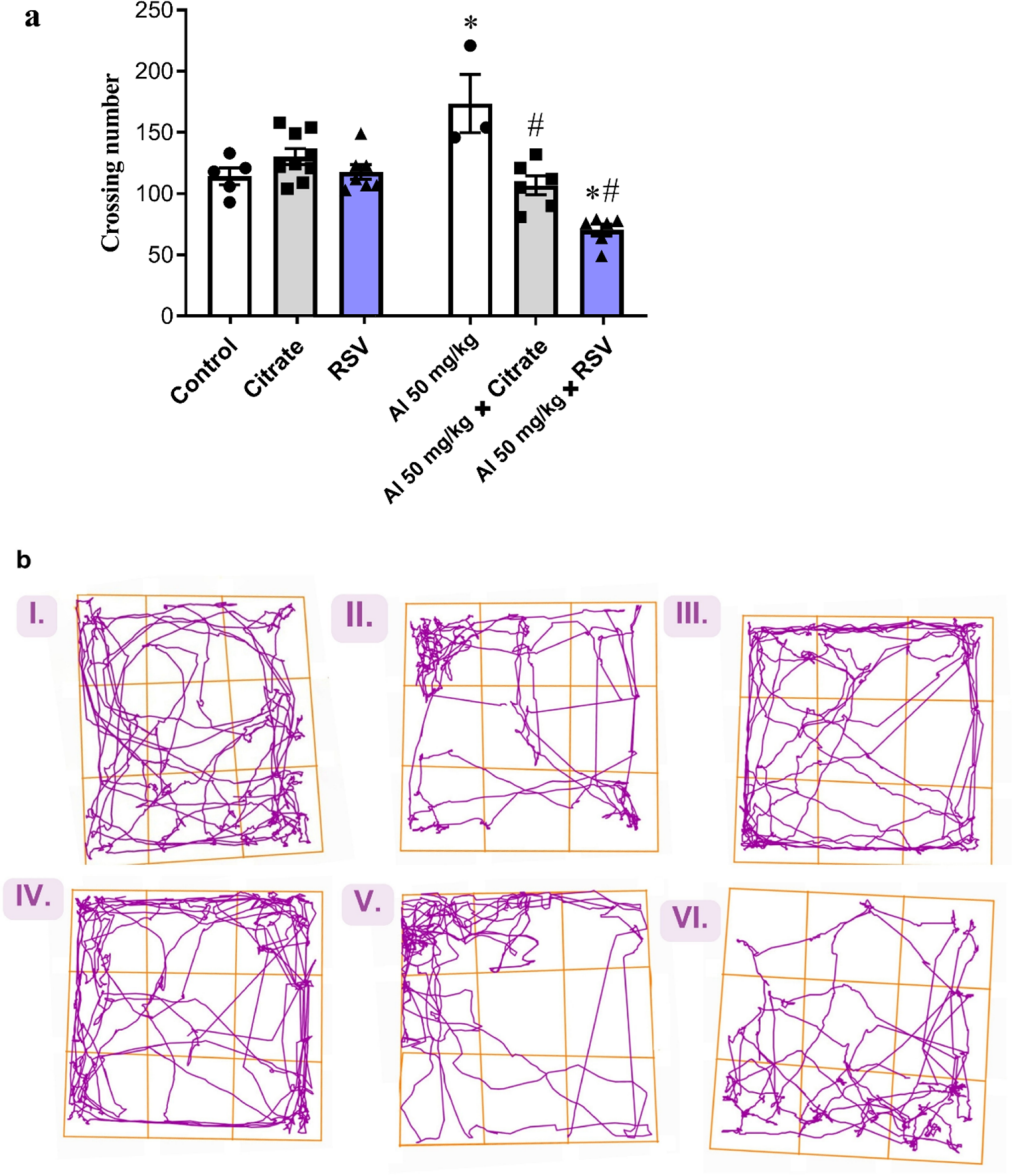
Exploratory activity in the Open Field Test (OFT)

In the Open Field Test (OFT; Fig. 2a), there were no significant differences in the number of crossings for the CIT (*p* = 0.6754) and RSV (*p* = 0.9996) groups compared with the Control group. In contrast, the AlCl_3_ group showed a significant increase (52.07%) in the number of crossings compared with the Control group (*p* = 0.0029). Furthermore, the AlCl_3_ + CIT and AlCl_3_ + RSV groups showed a significant reduction (38.48%, *p* = 0.0005 and 59.50%, *p* < 0.0001, respectively) compared with the AlCl_3_ group, and the AlCl_3_ + RSV group showed a significant reduction (38.41%; *p* = 0.0096) compared with the Control group. Figure 2b shows a representative trajectory plot of one animal from each experimental group. Overall, two-way ANOVA revealed a significant interaction between factors (F(2, 30) = 18.53, *p* < 0.0001) and a significant main effect of the column factor (F(2, 30) = 16.03, *p* < 0.0001), with no significant effect of the row factor (F(1, 30) = 0.31, *p* = 0.58).

This figure shows the effects of AlCl₃, CIT, and RSV treatments on spontaneous locomotor and exploratory behavior in male Swiss mice. Experimental groups included Control (*n* = 10), AlCl₃ (*n* = 9), CIT (*n* = 10), RSV (*n* = 9), AlCl₃+CIT (*n* = 8), and AlCl₃+RSV (*n* = 9). Animals excluded from analysis were due to technical errors during behavioral data processing. (a) Number of crossings recorded during the OFT, representing horizontal exploratory activity. Data are expressed as mean ± SEM and analyzed by two-way ANOVA followed by Tukey’s post hoc test. *p < 0.05 indicates significant differences compared to the Control group; #*p* < 0.05 compared to the AlCl₃ group; (b) Representative trajectories of a single mouse (*n* = 1) from each experimental group (I. Control, II. CIT, III. RSV, IV. AlCl₃, V. AlCl₃+CIT, VI. AlCl₃+RSV) illustrate the behavioral differences between treatments.

**Fig. 3:**
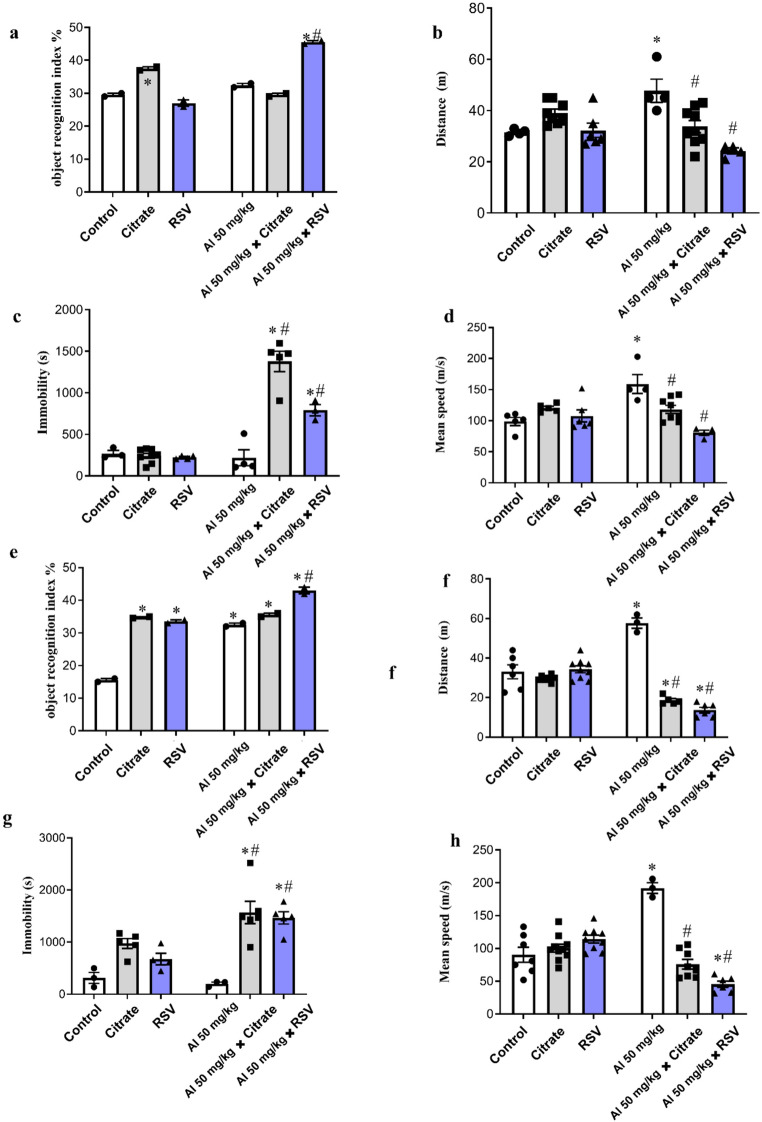
Protective effects of resveratrol on memory-related parameters and exploration behavior in male mice exposed to AlCl₃. This figure presents the behavioral outcomes from the Object Recognition Test (ORT) assessing short-term (4 h) and long-term (24 h) memory. Experimental groups included Control, AlCl₃, CIT, RSV, AlCl₃+CIT, and AlCl₃+RSV. (**a**) Short-term memory index; (**b**) Distance traveled (short memory phase); (**c**) Immobility time (short memory phase); (**d**) Mean speed (short memory phase); (**e**) Long-term memory index; (**f**) Distance traveled (long memory phase); (**g**) Immobility time (long memory phase); (**h**) Mean speed (long memory phase). Data are expressed as mean ± SEM (n = 2–10 animals per group) and analyzed by two-way ANOVA followed by Tukey’s post hoc test. *p < 0.05 compared to the Control group; #p < 0.05 compared to the AlCl₃ group

In the Object Recognition Test (ORT), when assessing short-term memory (after 4 h; Fig. 3a), the CIT and AlCl_3_ + RSV groups showed a significant increase (27.12%, *p* = 0.0008 and 54.24%, *p* < 0.0001, respectively) compared with the Control group. Furthermore, the AlCl_3_ + RSV group showed a significant increase (*p* < 0.0001) compared with both the AlCl_3_ and Control groups. Overall, two-way ANOVA for short-term memory revealed a significant interaction between factors (F(2, 6) = 225.5, *p* < 0.0001), as well as significant main effects of the row factor (F(1, 6) = 76.08, *p* = 0.0001) and the column factor (F(2, 6) = 35.72, *p* = 0.0005). When examining long-term memory (after 24 h; Fig. 3e), there was a significant increase in the CIT (124.19%), RSV (116.13%), AlCl_3_ (109.68%), AlCl_3_ + CIT (129.03%) and AlCl_3_ + RSV (177.42%) groups compared with the Control group (*p* < 0.0001). Furthermore, there was an increase in the AlCl_3_ + RSV (32.31%; *p* = 0.0001) groups compared with the AlCl_3_ group. Overall, two-way ANOVA for long-term memory revealed a significant interaction between factors (F(2, 6) = 96.21, *p* < 0.0001), as well as significant main effects of the row factor (F(1, 6) = 360.0, *p* < 0.0001) and the column factor (F(2, 6) = 326.4, *p* < 0.0001).

For the short-term memory locomotion analysis (after 4 h; Fig. 3b) the AlCl_3_ + RSV group covered a significantly shorter distance compared with the AlCl_3_ group (49.21%, *p* < 0.0001), while the AlCl_3_ + CIT group covered a significantly shorter distance (29.26%, *p* = 0.0067) compared with the AlCl_3_ group. Furthermore, the AlCl_3_ group covered a significantly longer distance (51.58%, *p* = 0.0075) compared with the Control group. Two-way ANOVA revealed a significant interaction between factors (F(2, 29) = 11.07, *p* = 0.0003) and a significant main effect of the column factor (F(2, 29) = 8.88, *p* = 0.0010), whereas no significant main effect of the row factor was observed (F(1, 29) = 0.19, *p* = 0.6646). In the long-term memory locomotion analysis (after 24 h; Fig. 3f), the CIT and RSV groups did not show significant difference in distance traveled compared with the Control group. The AlCl_3_ + CIT and AlCl_3_ + RSV groups showed a significant reduction compared with the Control group (43.68%, *p* = 0.0005 and 58.62%, *p* < 0.0001, respectively) and with the AlCl_3_ group (67.74%, *p* < 0.0001 and 76.30%, *p* < 0.0001, respectively), while the AlCl_3_ group showed a significant increase (74.60%, *p* < 0.0001) compared with the Control group. Two-way ANOVA revealed a significant interaction between factors (F(2, 30) = 56.42, *p* < 0.0001) and a significant main effect of the column factor (F(2, 30) = 57.41, *p* < 0.0001), whereas no significant main effect of the row factor was observed (F(1, 30) = 1.89, *p* = 0.1798).

The immobility time was assessed during the short-term memory test (after 4 h; Fig. 3c), and the AlCl₃+CIT and AlCl₃+RSV groups showed a significant increase in immobility time compared to the Control group (409.04%, *p* < 0.0001 and 192.24%, *p* = 0.0051, respectively) and to the AlCl₃ group (528.41%, *p* < 0.0001 and 260.77%, *p* = 0.0010, respectively). For immobility during the short-term memory phase, two-way ANOVA revealed a significant interaction between factors (F(2, 21) = 33.66, *p* < 0.0001), as well as significant main effects of the row factor (F(1, 21) = 77.94, *p* < 0.0001) and the column factor (F(2, 21) = 31.00, *p* < 0.0001). In the long-term memory test (after 24 h) (Fig. 3f), the AlCl₃+CIT and AlCl_3_+RSV groups also exhibited a significant increase in immobility time compared to the Control group (398.46%, *p* = 0.0003 and 365.82%, *p* = 0.0010, respectively) and to the AlCl₃ group (675.21%, *p* < 0.0001 and 624.45%, *p* = 0.0004, respectively). For immobility during the long-term memory phase, two-way ANOVA revealed a significant interaction between factors (F(2, 20) = 3.76, *p* = 0.0410), as well as significant main effects of the row factor (F(1, 20) = 10.62, *p* = 0.0039) and the column factor (F(2, 20) = 19.92, *p* < 0.0001).

Regarding the animals’ Mean speed, in the short-term memory test (Fig. 3d), the AlCl₃ group showed a significant increase compared to the Control group (60.96%, *p* = 0.0008), while the AlCl₃+CIT and AlCl₃+RSV groups exhibited a significant reduction compared to the AlCl₃ group (25.63%, *p* = 0.0171 and 49.21%, *p* < 0.0001, respectively). For the average speed during the short-term memory phase, two-way ANOVA revealed a significant interaction between factors (F(2, 26) = 13.16, *p* = 0.0001) and a significant main effect of the column factor (F(2, 26) = 8.61, *p* = 0.0014), whereas no significant main effect of the row factor was observed (F(1, 26) = 2.30, *p* = 0.1412). In the long-term memory test (Fig. 3h), the AlCl₃ group also showed a significant increase in mean speed compared to the Control group (112.32%, *p* < 0.0001), whereas the AlCl₃+RSV groups demonstrated a significant reduction compared to the Control group (49.68%, *p* = 0.0041) and to the AlCl₃ group (76.30%, *p* < 0.0001). In addition, the AlCl₃ + CIT group showed a significant reduction (60.48%, *p* < 0.0001) compared with the AlCl₃ group. For the average speed during the long-term memory phase, two-way ANOVA revealed a significant interaction between factors (F(2, 37) = 47.31, *p* < 0.0001) and a significant main effect of the column factor (F(2, 37) = 26.49, *p* < 0.0001), whereas no significant main effect of the row factor was observed (F(1, 37) = 0.17, *p* = 0.6822).

**Fig. 4:**
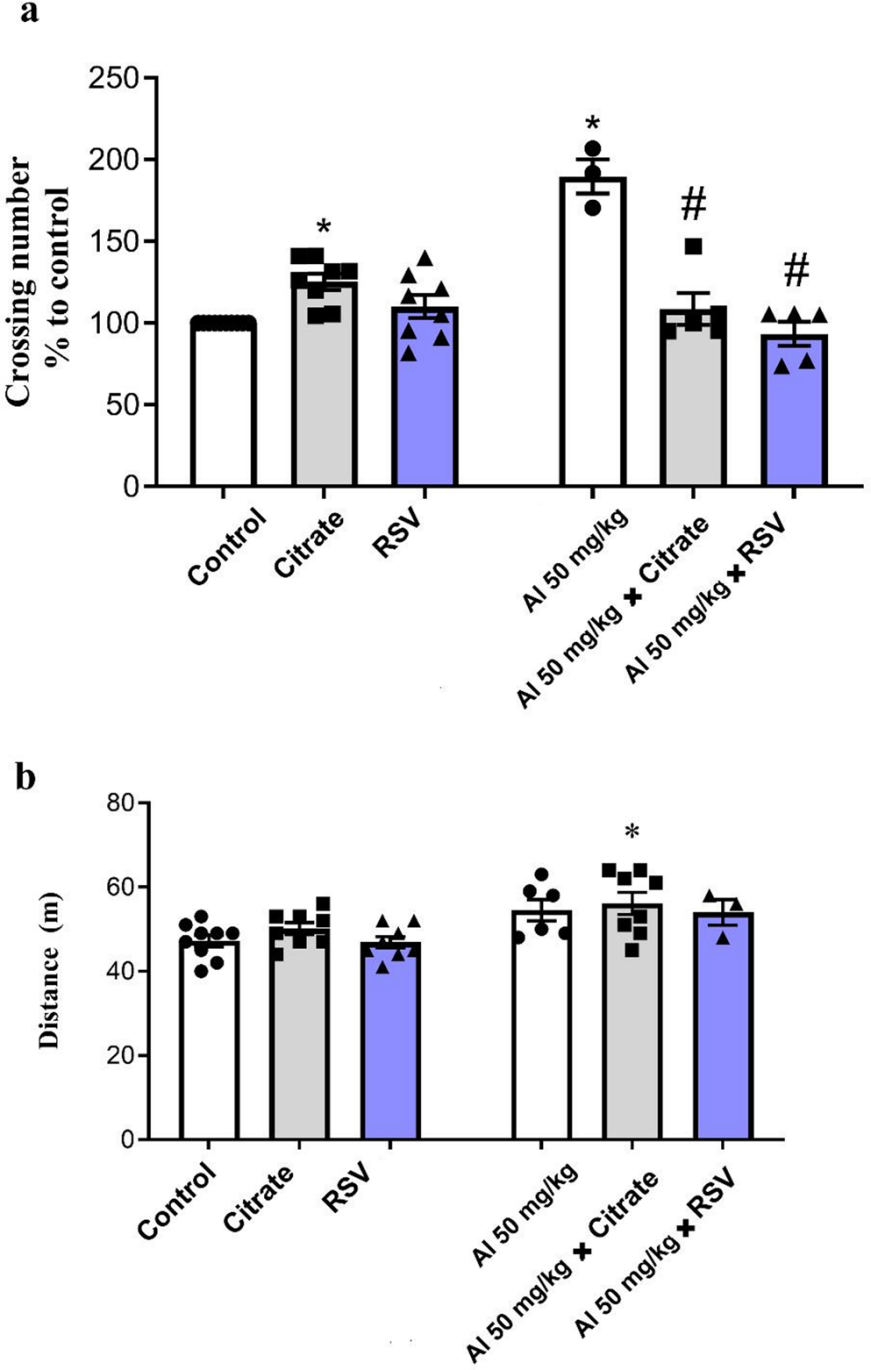
Resveratrol modulates spatial exploration and working memory behavior in male mice exposed to AlCl₃. This figure summarizes the performance of mice in the Y-Maze Test, which evaluates spontaneous alternation and locomotor activity as indicators of spatial working memory. Experimental groups were Control, AlCl₃, CIT, RSV, AlCl₃+CIT, and AlCl₃+RSV. (**a**) Percentage of spontaneous alternation; (**b**) Total distance traveled during the test. Data are shown as mean ± SEM (*n* = 3–10 animals per group) and analyzed by two-way ANOVA with Tukey’s post hoc test. *p < 0.05 vs. Control; #*p* < 0.05 vs. AlCl₃

In the Y-maze test (Fig. 4a), the CIT and AlCl_3_ groups showed a significant increase (25.21%, *p* = 0.0185 and 89.64%, *p* < 0.0001, respectively) compared with the Control group. In parallel, the AlCl_3_ + CIT and AlCl_3_ + RSV groups showed a significant reduction (42.73% and 50.68%, respectively; *p* < 0.0001) compared with the AlCl_3_ group. For the Y-maze test, two-way ANOVA revealed a significant interaction between factors (F(2, 33) = 39.52, *p* < 0.0001), as well as significant main effects of the row factor (F(1, 33) = 12.29, *p* = 0.0013) and the column factor (F(2, 33) = 20.55, *p* < 0.0001). In the Y-maze locomotion analysis (Fig. 4b), the distance covered by the AlCl_3_ + CIT group significantly increased (18.85%; *p* = 0.0162) compared with the Control group, while the other groups did not show a significant difference compared with the Control (*p* > 0.05). For the distance traveled in the Y-maze test, two-way ANOVA revealed no significant interaction between factors (F(2, 36) = 0.06, *p* = 0.9386) and no significant main effect of the column factor (F(2, 36) = 1.01, *p* = 0.3744), whereas a significant main effect of the row factor was observed (F(1, 36) = 15.08, *p* = 0.0004).

### RSV modulates ectonucleotidase and ADA activities in the cerebral cortex of male mice exposed to AlCl_3_.

**Fig. 5:**
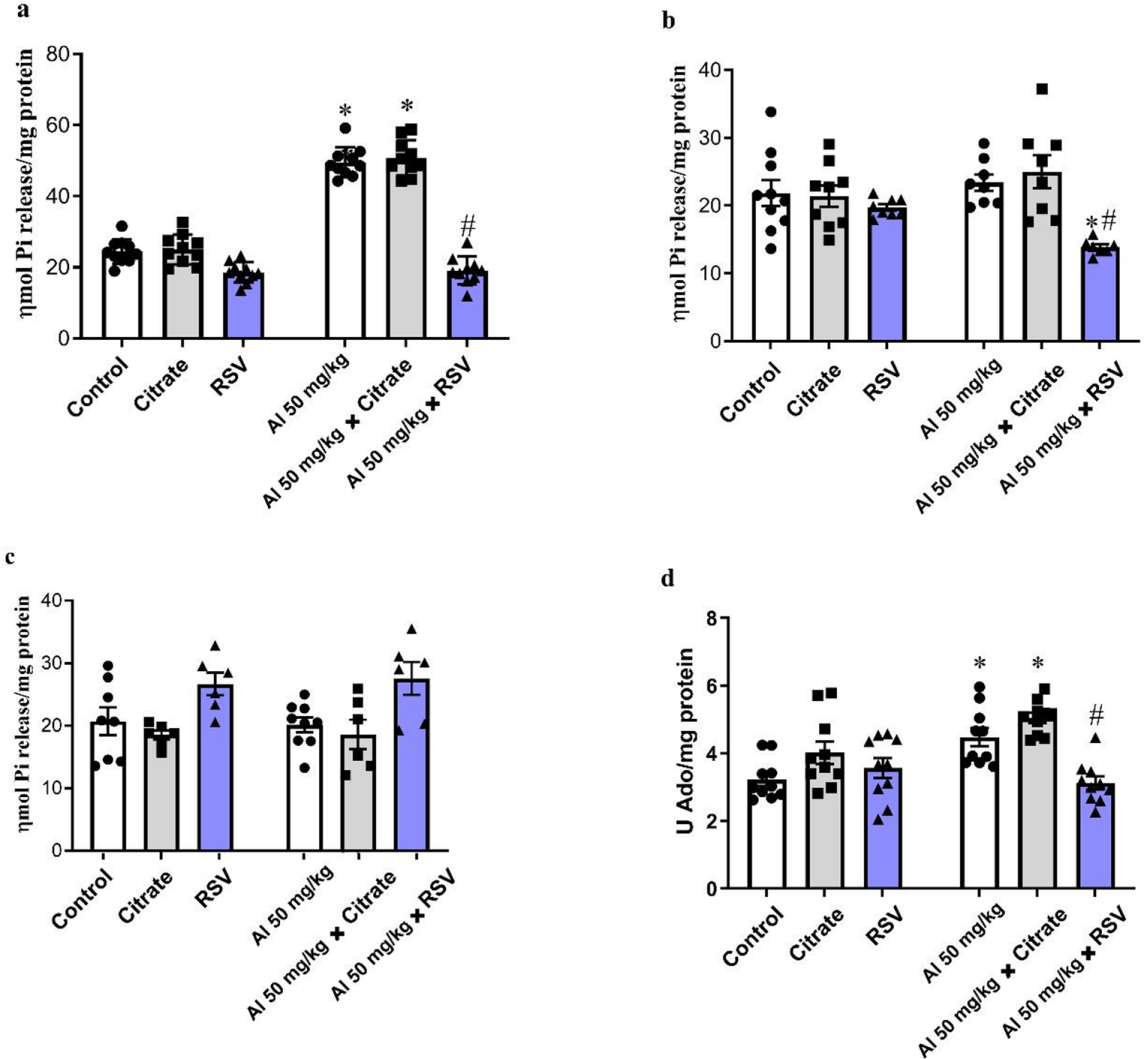
Effects of resveratrol on ectonucleotidase (NTPDase, 5’-NT) and adenosine deaminase (ADA) activities in the cerebral cortex of AlCl₃-exposed mice. This figure depicts the enzymatic activity of purinergic system components in the cerebral cortex of male Swiss mice after 30 days of treatment. Groups: Control, AlCl₃, CIT, RSV, AlCl₃+CIT, and AlCl₃+RSV. (**a**) ATP hydrolysis; (**b**) ADP hydrolysis; (**c**) AMP hydrolysis; (**d**) ADA activity (adenosine deamination). Data are expressed as mean ± SEM (*n* = 6–10 per group) and analyzed by two-way ANOVA followed by Tukey’s post hoc test. *p < 0.05 compared to Control; #*p* < 0.05 compared to AlCl₃. These results suggest that RSV and CIT treatments modulate purinergic enzyme activities, which may influence extracellular ATP and adenosine availability and thereby affect inflammatory tone and neuronal signaling processes associated with behavioral performance, potentially contributing to neuroprotection against aluminum toxicity

In the next step, it was investigated the hydrolysis of adenosine and its nucleoside in the cortex of mice exposed to RSV and/or AlCl_3_, given that extracellular ATP/adenosine balance is critically involved in neuroinflammatory signaling and synaptic modulation processes that may underlie the behavioral alterations observed in this study. The activities of NTPDase, 5’-NT, and ADA activities are shown in Fig. 5. In the cortex, ATP hydrolysis (Fig. 5a) by NTPDase was significantly increased in the AlCl_3_ and AlCl_3_ + CIT groups compared with the Control group (102.63% and 107.74%, respectively; *p* < 0.0001), and was significantly reduced in the AlCl_3_ + RSV group (61.46%) compared with the AlCl_3_ group (*p* < 0.0001). For ATP hydrolysis activity, two-way ANOVA revealed a significant interaction between factors (F(2, 54) = 64.76, *p* < 0.0001), as well as significant main effects of the row factor (F(1, 54) = 275.3, *p* < 0.0001) and the column factor (F(2, 54) = 144.9, *p* < 0.0001). ADP hydrolysis by NTPDase (Fig. 5b) showed a significant reduction in the AlCl_3_ + RSV group compared with the Control and AlCl_3_ groups (36.46%, *p* = 0.0116 and 40.65%, *p* = 0.0029, respectively). For ADP hydrolysis activity, two-way ANOVA revealed a significant interaction between factors (F(2, 44) = 4.64, *p* = 0.0148) and a significant main effect of the column factor (F(2, 44) = 9.39, *p* = 0.0004), whereas no significant main effect of the row factor was observed (F(1, 44) = 0.03, *p* = 0.8553). AMP hydrolysis by 5’-NT (Fig. 5c) was not different among the groups. For AMP hydrolysis activity, two-way ANOVA revealed no significant interaction between factors (F(2, 35) = 0.08, *p* = 0.9253) and no significant main effect of the row factor (F(1, 35) = 0.01, *p* = 0.9353), whereas a significant main effect of the column factor was observed (F(2, 35) = 10.13, *p* = 0.0003). Regarding ADA activity, measured through ADO deamination (Fig. 5d) the AlCl_3_ and AlCl_3_ + CIT groups showed a significant increase compared with the Control group (39.01%, *p* = 0.0085 and 56.63%, *p* < 0.0001, respectively; *p* < 0.05). Furthermore, the AlCl_3_ + RSV group showed a significant reduction compared with the AlCl_3_ group (30.34%; *p* < 0.05). For adenosine deaminase (ADA) activity in the cerebral cortex, two-way ANOVA revealed a significant interaction between factors (F(2, 54) = 7.01, *p* = 0.0020), as well as significant main effects of the row factor (F(1, 54) = 9.31, *p* = 0.0035) and the column factor (F(2, 54) = 11.75, *p* < 0.0001).

### RSV modulates purinergic receptors and increases BDNF levels in male mice exposed AlCl_3_

**Fig. 6:**
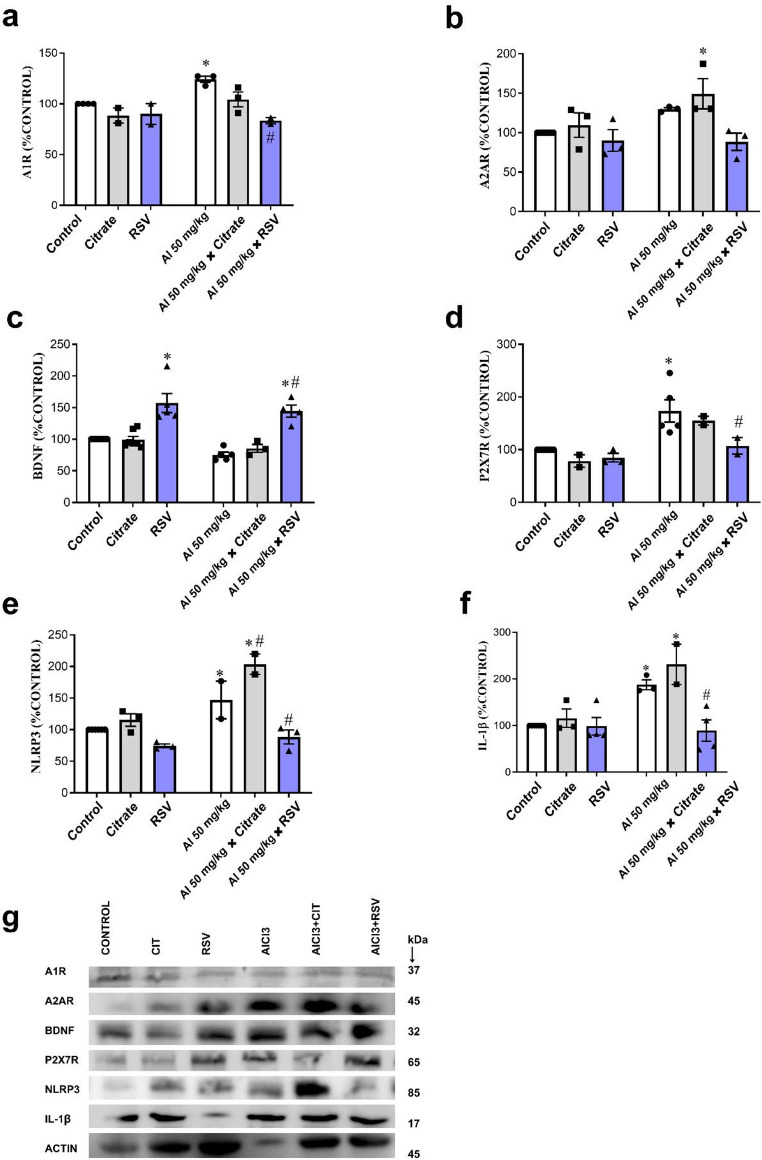
Protein density of purinoreceptors, an inflammatory protein, and a neurotrophin in the cerebral cortex of AlCl3-exposed male Swiss mice. This figure illustrates Western blot analysis of protein expression in the cerebral cortex of male Swiss mice. Groups: Control, AlCl₃, CIT, RSV, AlCl₃+CIT, and AlCl₃+RSV. (**a**) A1R; (**b**) A2AR; (**c**) BDNF; (**d**) P2 × 7R; (**e**) NLRP3; (f) IL-1β; (**g**) Representative blots showing protein bands for each group. Data are shown as mean ± SEM (*n* = 2–7 animals per group) and analyzed by two-way ANOVA followed by Tukey’s post hoc test. *p < 0.05 vs. Control; #*p* < 0.05 vs. AlCl₃. The figure demonstrates that RSV treatment modulated both purinergic receptor expression and inflammatory markers, suggesting a regulatory effect on neuroinflammation and neurotrophic signaling

Because AlCl_3_ and RSV modulated the activities of ectoenzymes in the cortex of mice, we also investigated whether these compounds could alter the density of the purinoreceptors P2 × 7R, A1R, and A2AR, as well as IL-1β, NLRP3 and BDNF with Western blot (Fig. 6). The corresponding full, uncropped Western blot membranes are provided in the Supplementary Material (Figs. S1–S7). There was a significant reduction in A1R in the AlCl_3_ + RSV group compared with the AlCl_3_ group (32.75%, *p* = 0.0036), while there was a significant increase in A1R in the AlCl_3_ group compared with the Control group (24.05%; *p* = 0.0338). Moreover, the other groups did not show a significant variation in A1R receptor density compared to the Control group and AlCl_3_ group (Fig. 6a). For adenosine A1 receptor protein levels, two-way ANOVA revealed a significant interaction between factors (F(2, 10) = 4.22, *p* = 0.0468), as well as significant main effects of the row factor (F(1, 10) = 6.48, *p* = 0.0291) and the column factor (F(2, 10) = 12.27, *p* = 0.0020). Regarding A2AR, only the AlCl₃+CIT group showed a significant increase in density (49.29%, *p* < 0.0036) compared to the Control group (Fig. 6b). For adenosine A2A receptor protein levels, two-way ANOVA revealed no significant interaction between factors (F(2, 19) = 2.49, *p* = 0.1098), whereas significant main effects of the row factor (F(1, 19) = 8.93, *p* = 0.0075) and the column factor (F(2, 19) = 8.59, *p* = 0.0022) were observed. The BDNF increased significantly in the AlCl_3_ + RSV group compared with the AlCl_3_ group (91.05%, *p* < 0.0001) and the Control group (44.43%; *p* = 0.0009). Furthermore, the RSV group showed a increased significantly (57.23%; *p* < 0.0001) compared with the Control group. The other groups did not show a significant variation in BDNF density compared to the Control group and AlCl_3_ group (*p* > 0.05; Fig. 6c). For brain-derived neurotrophic factor (BDNF) protein levels, two-way ANOVA revealed no significant interaction between factors (F(2, 28) = 0.45, *p* = 0.6431), whereas significant main effects of the row factor (F(1, 28) = 8.19, *p* = 0.0079) and the column factor (F(2, 28) = 45.79, *p* < 0.0001) were observed. The P2 × 7R levels increased significantly in the AlCl_3_ group compared with the Control group (73.64%, *p* = 0.0003) and decreased significantly in the AlCl_3_ + RSV group compared with the AlCl_3_ group (38.26%; *p* < 0.0376). The other groups did not show a significant variation in P2 × 7R density compared to the Control group and AlCl_3_ group (*p* > 0.05; Fig. 6d). For P2 × 7 receptor protein levels, two-way ANOVA revealed no significant interaction between factors (F(2, 18) = 2.22, *p* = 0.1380), whereas significant main effects of the row factor (F(1, 18) = 24.48, *p* = 0.0001) and the column factor (F(2, 18) = 5.40, *p* = 0.0146) were observed. The NLRP3 increased significantly in the AlCl_3_ group (46.93%, *p* = 0.0416) compared with the Control group and in the AlCl_3_ + CIT group compared with the Control group and the AlCl_3_ group (103.52%, *p* < 0.0001 and 38.51%, *p* = 0.0456, respectively). Furthermore, in the AlCl_3_ + RSV group, there was a significant reduction (39.82%; *p* < 0.0208) compared with the AlCl_3_ group (Fig. 6e). For NLRP3 protein levels, two-way ANOVA revealed a significant interaction between factors (F(2, 13) = 6.53, *p* = 0.0109), as well as significant main effects of the row factor (F(1, 13) = 36.41, *p* < 0.0001) and the column factor (F(2, 13) = 29.07, *p* < 0.0001). Finally, the IL-1β showed a significant increase in the AlCl_3_ group and AlCl_3_ + CIT group (80.64%, *p* = 0.0097 and 131.48%, *p* = 0.0009, respectively) compared with the Control group. Furthermore, the AlCl_3_ + RSV group showed a significant increase compared with the AlCl_3_ group (52.36%, *p* = 0.0087; Fig. 6f). For IL-1β protein levels, two-way ANOVA revealed a significant interaction between factors (F(2, 17) = 7.47, *p* = 0.0047), as well as significant main effects of the row factor (F(1, 17) = 20.78, *p* = 0.0003) and the column factor (F(2, 17) = 10.49, *p* = 0.0011). It is possible to observe in Fig. 6g the bands from the Western blot readings of the analyzed signaling molecules. Taken together, our findings suggest that Al^3+^ exerts neuroinflammation and RSV exerts neuroprotection, with changes in purinergic receptor expression (A1R, A2AR, and P2 × 7R) providing a mechanistic link between inflammatory signaling and the behavioral alterations observed in aluminum-exposed mice.

## Discussion

Al^3+^ is ubiquitous in the environment, and as a result, humans are constantly exposed to this metal.

Chronic exposure to Al³⁺ has been associated with cognitive impairments and dementia (Kawahara et al. 2021). In view of this, the present in vivo study was conducted to determine whether exposure to AlCl₃ at a concentration of 50 mg/kg body weight affects behavior, the purinergic system, and inflammatory signaling in Swiss mice. The experimental design incorporated aluminum–citrate interactions to characterize how citrate modulates aluminum-related toxicity and behavioral outcomes, given its known ability to enhance aluminum absorption and biodistribution. However, aluminum concentrations were not directly quantified in biological matrices, similar to other experimental studies employing aluminum exposure models combined with citrate or relying on well-established behavioral and molecular endpoints without direct tissue aluminum measurement (Nolan et al. 1990; Wu et al. 2012; Al-Amin et al. 2016). In addition, although citrate was included as a modulator of aluminum toxicity, the absence of a combined AlCl₃ + CIT + RSV group precludes direct assessment of whether resveratrol maintains its neuroprotective effects under conditions of citrate-enhanced aluminum bioavailability. Thus, conclusions regarding RSV are confined to its effects in the context of aluminum exposure without concomitant citrate modulation. Therefore, the findings should be interpreted as functional and molecular outcomes associated with aluminum exposure rather than as direct measures of tissue aluminum burden. More specifically, the study aimed to investigate whether resveratrol (RSV), a potent natural antioxidant, could mitigate the cognitive deficits and neuroinflammatory alterations induced by Al³⁺ exposure.

The results show that mice treated with AlCl₃ exhibited signs consistent with Al³⁺ intoxication, including alterations in general behavior such as bristly hair, excessive grooming, and changes in activity patterns. In the open-field test (OFT), AlCl₃-treated mice displayed higher locomotor and exploratory activity. Although the OFT is not a specific assay for anxiety-related behavior, changes in locomotion and exploration may reflect alterations in behavioral activation, arousal state, or stress responsiveness induced by Al³⁺ exposure. Depending on the context, such behavioral changes may manifest as either increased agitation or reduced activity (Frederiksen and Waldemar 2017; Ma 2020). These observations align with prior reports showing that Al³⁺ exposure can increase exploratory activity in open-field paradigms (Al-Amin et al. 2016; Capriello et al. 2021), supporting an interpretation centered on altered locomotor and exploratory behavior rather than a specific anxiety phenotype. The object recognition test (ORT) primarily indexes recognition memory. In the AlCl₃+RSV group, the discrimination index increased while locomotion and mean speed declined. Overall exploration remained sufficient, indicating that reduced locomotion did not confound memory performance. Thus, the improvement in the discrimination index under RSV treatment is interpreted as enhanced recognition memory. Apparent changes in ORT performance in AlCl₃-exposed mice are more plausibly explained by alterations in novelty processing, object salience, or exploratory strategy. Regarding behavioral differences between the AlCl₃ and AlCl₃+CIT groups, citrate is known to alter aluminum toxicokinetics, including increased bioavailability via soluble complexes. In our data, the addition of CIT modified, rather than uniformly exacerbated, Al³⁺-related phenotypes, yielding shifts in exploratory behavior. We therefore describe CIT as modulating Al³⁺ toxicity and behavioral expression.

In the Y-maze test, the AlCl₃ group showed an increased number of crossings, which may reflect heightened exploratory drive, altered locomotor activity, or changes in behavioral organization. Increased arm entries in the Y-maze have been associated with altered arousal states or impaired behavioral inhibition in neurotoxicity models. Similarly, in the AlCl₃+CIT group, increased activity in the Y-maze suggests modulation of exploratory behavior by citrate.

It has been reported that RSV and Al^3+^ can modulate specific proteins, including enzymes that hydrolyze extracellular ATP, ADP, AMP and ADA activity (Bottari et al. 2019; Zhu et al. 2023). Furthermore, Cauwels et al. (2014), showed that removing extracellular ATP prevented the production of inflammasome-independent cytokines such as tumor necrosis factor alpha (TNF-α). Cunha (2016) observed that a large flow of ATP is triggered by brain damage as a danger signal. It has also been shown that ADA influences neuronal plasticity, including learning and memory processes, and plays an important role in neuroinflammation (Garcia-Gil et al. 2021; Chang et al. 2021). Huang et al. (2019) observed that ADA suppression had beneficial effects in a Parkinson’s mouse model and other neurodegenerative disorders. The significant increase in ATP hydrolysis by NTPDase and in ADA activity in the AlCl₃ and AlCl₃ + CIT groups supports the pro-inflammatory mechanism linked to Al^3+^ exposure, which is relevant in the context of the behavioral alterations observed, since excessive purinergic signaling is known to disrupt synaptic function, neuronal excitability, and cognitive processing. Extracellular ATP, when converted into ADO by the ADA enzyme, can activate purinergic receptors, amplifying the inflammatory response and stress, which reflects the intensification of inflammation. On the other hand, RSV, due to its antioxidant and anti-inflammatory properties, may mitigate the deleterious effects of Al³^+^ on ATP and adenosine metabolism. The reduction in ATP and ADA activity observed in the AlCl₃+RSV group suggests that RSV exerts a modulatory effect, decreasing the excessive release of ATP and its subsequent conversion into ADO. This effect may be attributed to RSV’s ability to neutralize free radicals and reduce oxidative stress, thereby limiting excessive activation of the inflammatory response and, consequently, the need for large amounts of ATP and ADO as signals of cellular damage. These results are in line with previous studies showing the interaction of the purinergic system with Al³+, suggesting that modulation of ATP hydrolysis by NTPDase may be a relevant mechanism for mediating the pro-inflammatory effects of Al³^+^ (Cieślak and Wojtczak 2018; Reichert et al. 2020; Assmann et al. 2021). Thus, the observed reduction in the AlCl₃ + RSV group reflects the ability of resveratrol to attenuate purinergic system activation, creating a less inflammatory environment, which reinforces the protective effect of the antioxidant against aluminum-induced damage (Bottari et al. 2019).

In pathological conditions, adenosine plays a protective role by modulating the release of neurotransmitters and binding to specific receptors (Braga et al. 2019). The increased levels of A1R in the AlCl₃ group and A2AR in the AlCl₃+CIT group suggest that these receptors are involved in cognitive impairment. It is believed that these receptors are positively modulated during Al³+-induced neuroinflammation. Although A1R has already been described as neuroprotective, its role in neurodegeneration has not yet been elucidated. The study by Stockwell et al. (2017) clarifies that excessive activation of A1R and A2AR receptors can lead to undesirable effects. Giunta et al. (2014) found that dual blockade of the adenosine receptors A1R and A2AR prevented beta-amyloid toxicity in neuroblastoma cells exposed to aluminum chloride. This mechanism may parallel the neural damage observed in Al³⁺ intoxication, suggesting potential overlaps in the pathological pathways. The Western blot results indicated that the density of the receptor was reduced in the AlCl₃ + RSV group compared to the AlCl₃ group. Therefore, modulating A1R levels may represent a promising strategy for the treatment of dementia (Stockwell et al. 2017). Previous studies have observed that the selective blockade of A1R and A2AR facilitates learning and memory in rodents in behavioral tests (Cunha, 2008; Pagnussat et al. 2015; Mouro et al. 2017). Rodríguez et al. (2006) demonstrated an upregulation and sensitization of A1R and A2AR in the frontal cortex in AD as an early event in disease progression, findings that align with the observed effects of Al^3+^ intoxication reported in the present study. The involvement of A1R and A2AR in previous studies supports the findings regarding Al^3+^ intoxication, indicating that these receptors play a role in the inflammatory mechanism triggered by Al^3+^. Similarly, the results suggest that RSV mitigates these processes, reducing inflammation and providing neuroprotection during Al^3+^ intoxication.

Regarding the P2 × 7R, it is activated by high levels of ATP, so the increase in the density of this receptor observed in the cortex of mice treated with Al^+ 3^ could be part of the inflammation resulting from intoxication (Zarrinmayeh and Territo 2020). This mechanism is possibly part of the processes that involve the purinergic cascade of inflammation which culminates in emergence of AD (Illes et al. 2019). Activation of the P2 × 7R receptor observed in the AlCl₃ group is associated with the development of a series of intracellular events, including the release of pro-inflammatory cytokines, such as TNF-α, and the formation of pores in the cell membrane. This allows the entry of calcium and other ions, resulting in cellular damage. On the other hand, the negative modulation of this receptor in the AlCl_3_ + RSV group corroborates the hypothesis that RSV counteracts the damage arising by Al^3+^ intoxication. Thus, RSV appears to modulate P2 × 7R activation and delay the inflammatory cascade triggered by Al^3+^ intoxication, which may reduce neuroinflammation-driven synaptic dysfunction and thereby contribute to the improvement of cognitive and exploratory behaviors observed in RSV-treated animals, as also demonstrated in the study by Thawkar and Kaur (2019).

The levels of the inflammasome NLRP3 and the inflammatory cytokine IL-1β were upregulated in the AlCl₃ and AlCl₃+CIT groups, indicating the activation and amplification of neuroinflammatory signaling cascades (Zhu et al. 2023). In contrast, the density of this protein was reduced in the AlCl₃ + RSV group, suggest that RSV interferes with inflammasome priming and/or activation, thereby exerting a neuroprotective effect. Current evidence indicates that NLRP3 activation is a two-step process involving an initial priming phase largely mediated by NF-κB–dependent transcription of inflammasome components followed by an activation phase triggered by mitochondrial dysfunction, excessive reactive oxygen species (ROS) production, potassium efflux, and extracellular ATP accumulation. These signals converge to promote caspase-1 activation and the proteolytic maturation of IL-1β, thereby amplifying neuroinflammation, synaptic dysfunction, and neuronal damage (Zhu et al. 2020). Recent high-quality studies demonstrate that bioactive natural compounds, including polyphenols, attenuate NLRP3-driven neuroinflammation by preserving mitochondrial integrity, suppressing NF-κB signaling, and limiting ROS-dependent inflammasome activation, ultimately reducing IL-1β release and pyroptotic cell death (Qian et al. 2023; Rong et al. 2025). These mechanisms establish a direct causal link between inflammasome activation, synaptic dysfunction, and cognitive impairment, rather than viewing inflammation as a secondary epiphenomenon. In line with this framework, inhibition of NLRP3 signaling has been consistently associated with improved cognitive outcomes and reduced neurodegenerative burden in experimental models (Venegas et al. 2017; Ising et al. 2019; Stancu et al. 2019). Therefore, the downregulation of NLRP3 and IL-1β observed following resveratrol treatment supports a mechanistic model in which modulation of mitochondrial and NF-κB–dependent inflammasome signaling underlies the protective effects of RSV against Al³⁺-induced neurotoxicity.

Neurotrophins, such as BDNF, are essential for neuronal plasticity and neuroprotection. dysregulation of BDNF has been associated with various diseases, including progression to AD (Tanila 2017). This neurotrophin, abundantly expressed in brain regions with high plasticity, such as the hippocampus, hypothalamus, and cortex (Huang and Reichardt, 2001), was positively modulated by RSV during Al³+ intoxication, suggesting that RSV plays a role in promoting neuronal longevity. In contrast, the AlCl3 and AlCl3 + CIT groups showed a non-significant reduction in BDNF levels, which may indicate an early stage of neuronal damage. BDNF is responsible for regulating synaptic transmission and its level is regulated by neuronal activity (Tapia-Arancibia et al. 2008). Koo et al. (2013) observed that BDNF levels decrease in patients with AD, and this reduction results in impaired spatial learning and memory, which is consistent with the findings of this study. In addition, BDNF can induce an increase in somatostatin, which in turn activates neprilysin, an enzyme responsible for the degradation of Aβ in the brain. This process helps reduce Aβ accumulation, which is toxic to neurons and associated with neurodegenerative diseases such as AD. Thus, the increase in BDNF may exert a neuroprotective effect, helping to prevent neurodegeneration and improve cognitive function. Mechanistically, Al³⁺-driven purinergic signaling (e.g., ATP→P2 × 7R) can engage the NLRP3 inflammasome, elevating IL-1β and fostering synaptic dysfunction. Conversely, BDNF supports synaptic plasticity; increases in BDNF observed with RSV may counteract NLRP3-linked damage. This NLRP3–BDNF interplay provides a plausible mechanistic framework linking inflammation and cognition in the present model and may underlie the neuroprotective actions of RSV during Al³⁺ intoxication.

## Conclusion

Exposure to Al^3+^ caused impairment of short- and long-term memory in male Swiss mice, with the addition of CIT seemingly exacerbating this condition. Furthermore, Al^3+^ induced an inflammatory effect, evidenced by the modulation of inflammatory nucleotides and the nucleoside ADA, in addition to positively stimulating inflammatory purinergic receptors. In parallel, treatment with RSV mitigated the effects of the anxiogenic response triggered by Al^3+^ intoxication, modulated the hydrolysis of inflammatory nucleotides in the cortex, and increased BDNF, a neurotrophin associated with neuronal survival, while also negatively modulating inflammation-related receptors. In summary, this study highlights the crucial role of the purinergic system during Al^3+^ exposure and suggests that RSV may be a promising therapy to reduce Al^3+^-induced cognitive deficits.

## Supplementary information

Below is the link to the electronic supplementary material.

Supplementary Material 1 (XLSX 21.7 KB)

Supplementary Material 2 (DOCX 1.12 MB)

## Data Availability

The datasets generated and/or analyzed during the current study are not publicly available due to their large volume and non-standardized format, but are available from the corresponding author upon reasonable request.
