# Supplementary material for: Resveratrol as a potential natural compound to ameliorate cognitive impairment in aluminum-exposed mice: impacts on behavior, purinergic system, and brain inflammation
Source: Metab Brain Dis. 2026 May 19;41(1):96. doi: 10.1007/s11011-026-01859-z (PMC13183731; doi:10.1007/s11011-026-01859-z)
Supplement: Supplementary file 2 — Supplementary Material 2 (DOCX 1.12 MB) [file 11011_2026_1859_MOESM2_ESM.docx]

**RESVERATROL AS A POTENTIAL NATURAL COMPOUND TO AMELIORATE COGNITIVE IMPAIRMENT IN ALUMINUM-EXPOSED MICE: IMPACTS ON BEHAVIOR, PURINERGIC SYSTEM, AND BRAIN INFLAMMATION**

Alice Estivalet Visentini^a,b*^; Karine Paula Reichert^a^; Maria Rosa Chitolina Schetinger^a^; Nathieli Bianchin Bottari^a^; Vanessa Valéria Mirona^a^; Milagros Fanny Vera Castro^a^, Marcylene Vieira da Silveira^a^; Charles Elias Assmann^a^, Adriel Antonio Schirmann^a^, Vera Maria Melchiors Morsch^a,b*^

**Supplementary information**


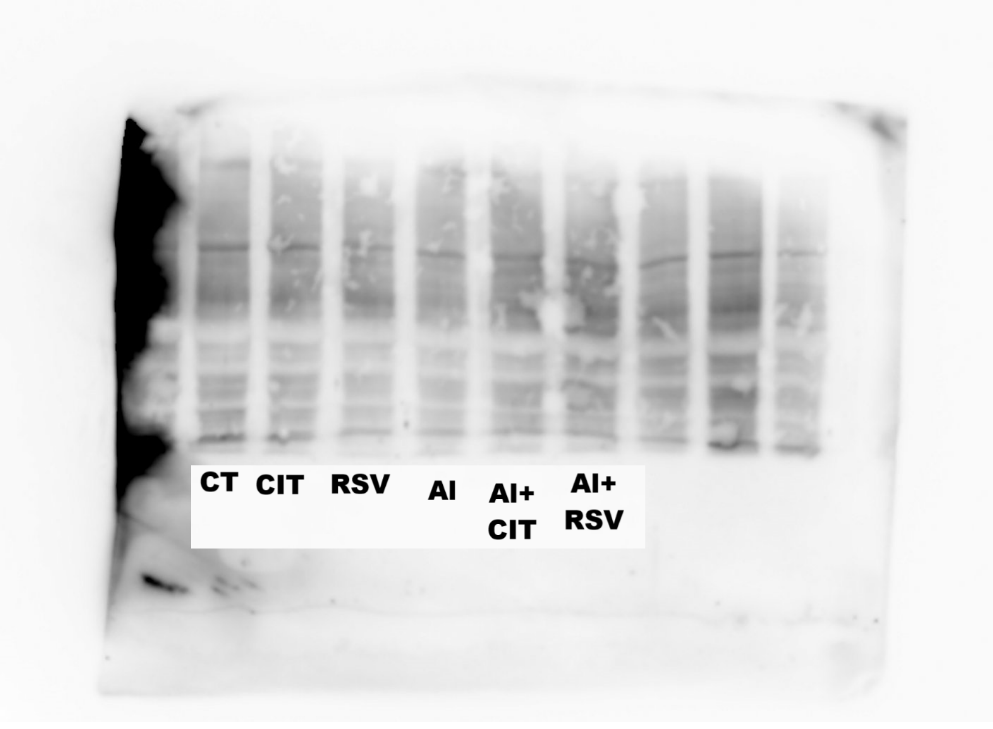


**Fig. S1 Representative full-length Western blot membrane for the adenosine A1 receptor (A1R)**

The bands correspond to cerebral cortex samples from male Swiss mice subjected to the different experimental treatments.


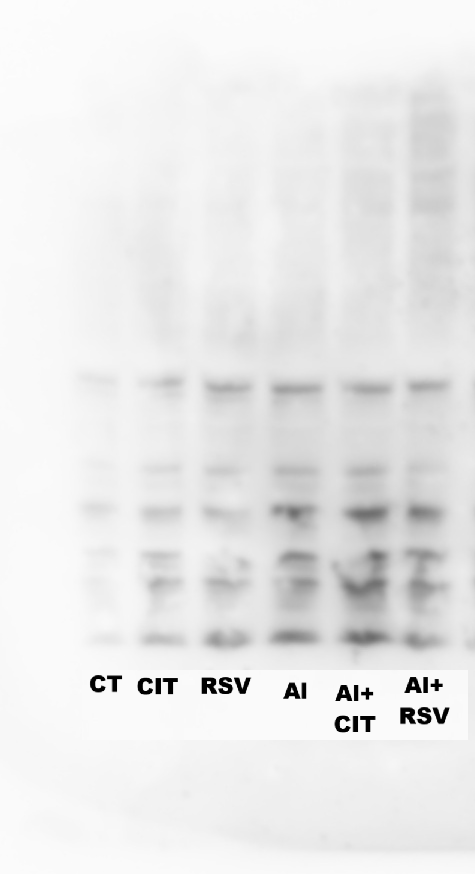


**Fig. S2 Representative full-length Western blot membrane for the adenosine A2A receptor (A2AR)**

The bands correspond to cerebral cortex samples from male Swiss mice subjected to the different experimental treatments.


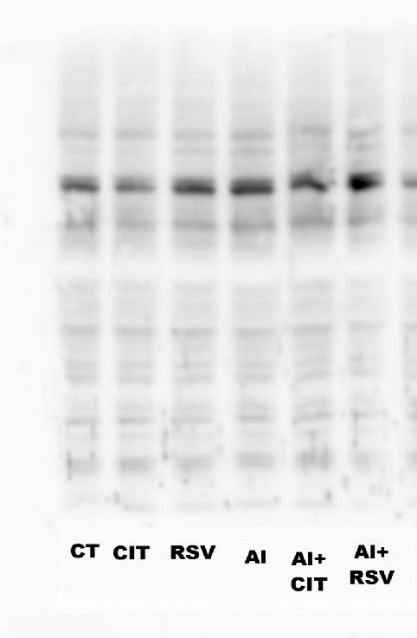


**Fig. S3 Representative full-length Western blot membrane for brain-derived neurotrophic factor (BDNF)**

The bands correspond to cerebral cortex samples from male Swiss mice subjected to the different experimental treatments.

**
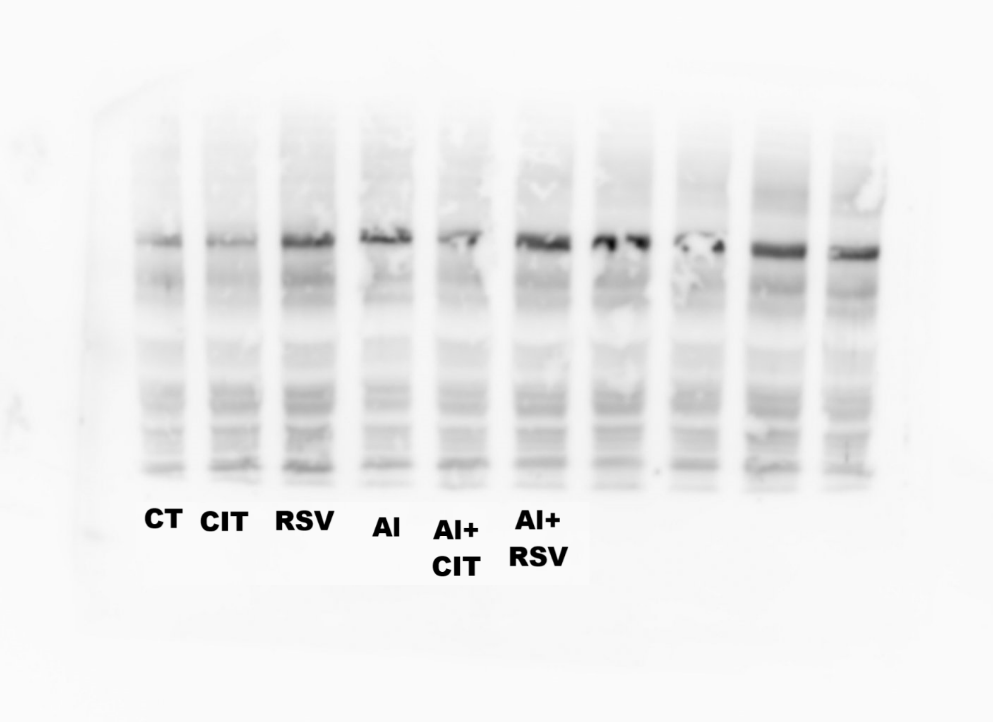
**

**Fig. S4 Representative full-length Western blot membrane for the P2X7 receptor (P2X7R)**

The bands correspond to cerebral cortex samples from male Swiss mice subjected to the different experimental treatments.


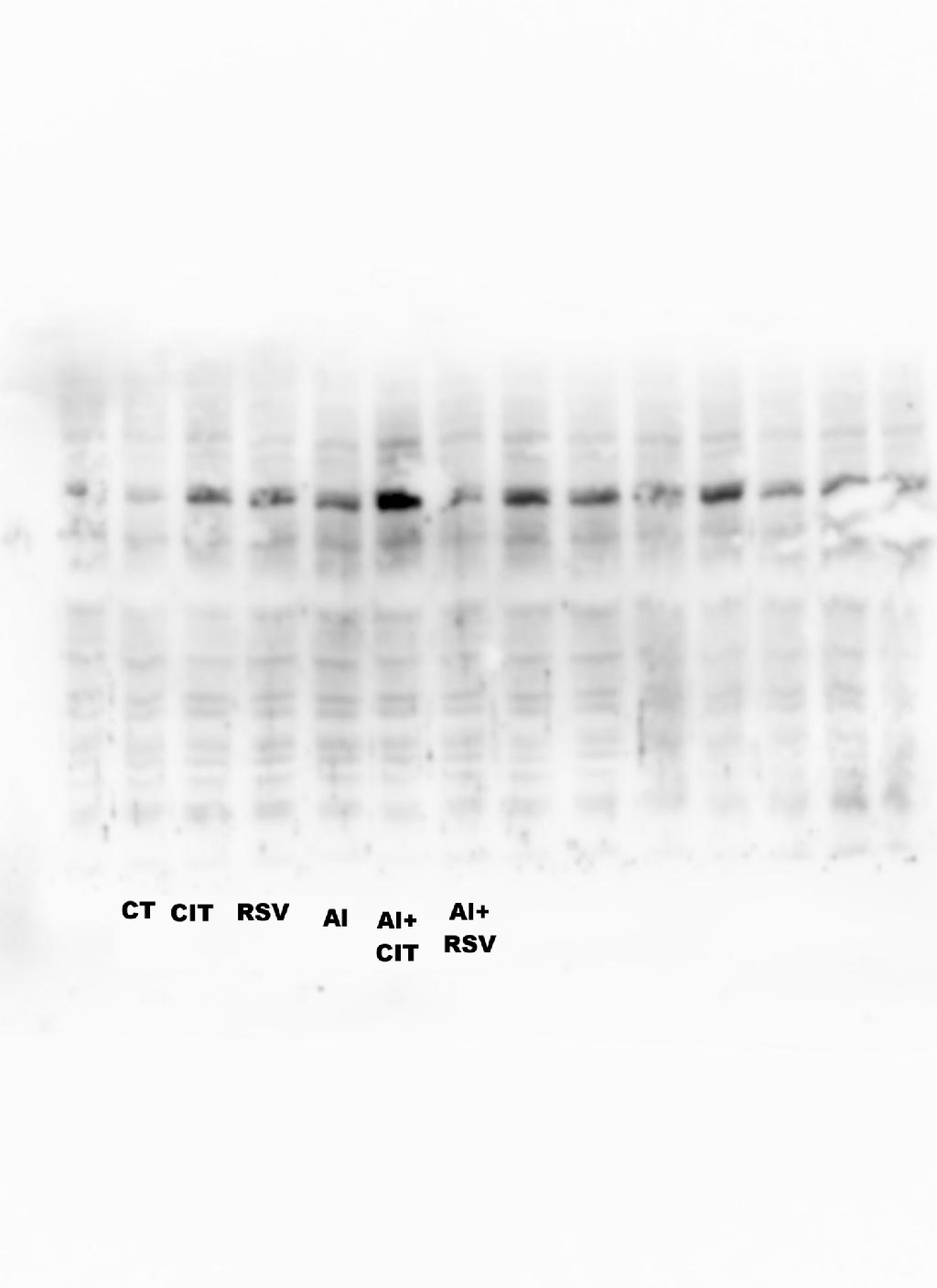


**Fig. S5 Representative full-length Western blot membrane for the NLRP3 inflammasome**

The bands correspond to cerebral cortex samples from male Swiss mice subjected to the different experimental treatments.


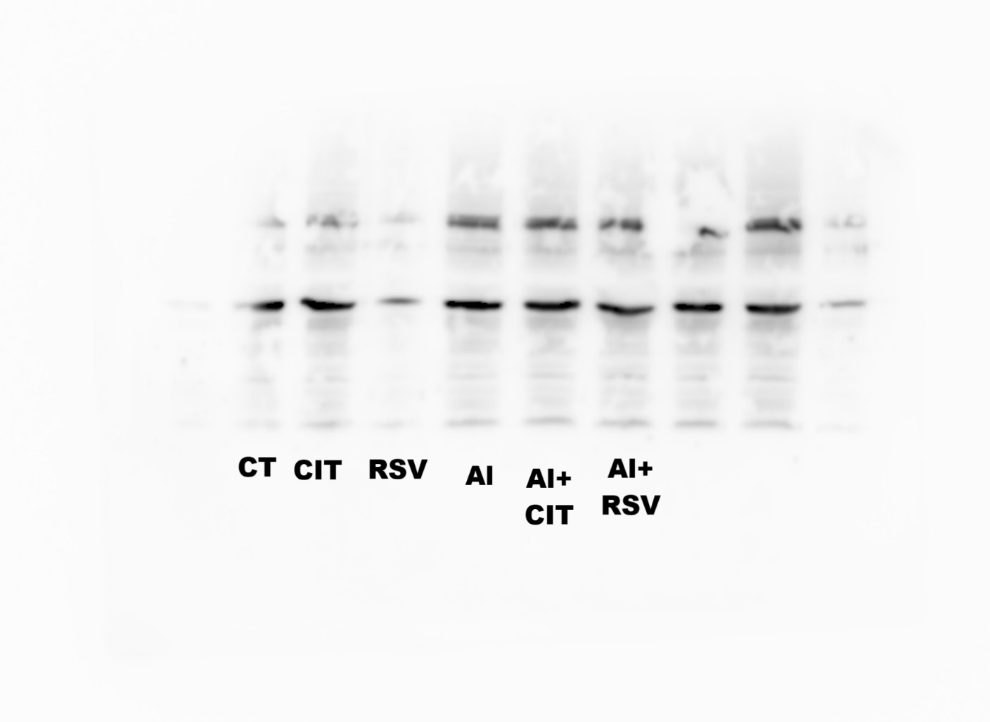


**Fig. S6 Representative full-length Western blot membrane for interleukin-1 beta (IL-1β)**

The bands correspond to cerebral cortex samples from male Swiss mice subjected to the different experimental treatments.


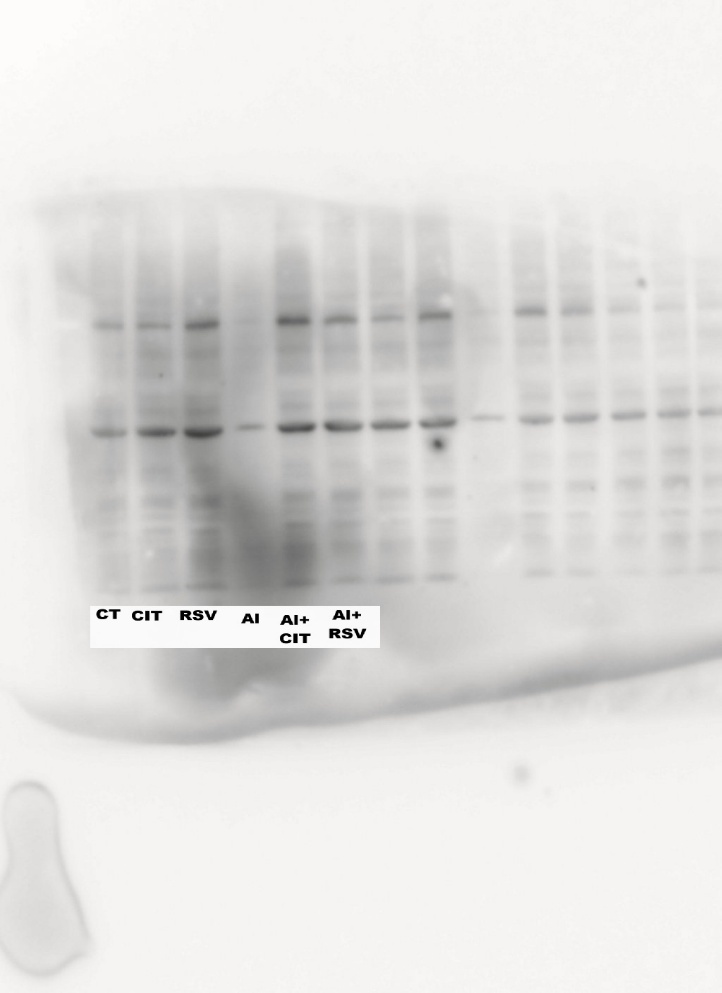


**Fig. S7 Representative full-length Western blot membrane for β-actin**

β-Actin was used as the internal loading control.
